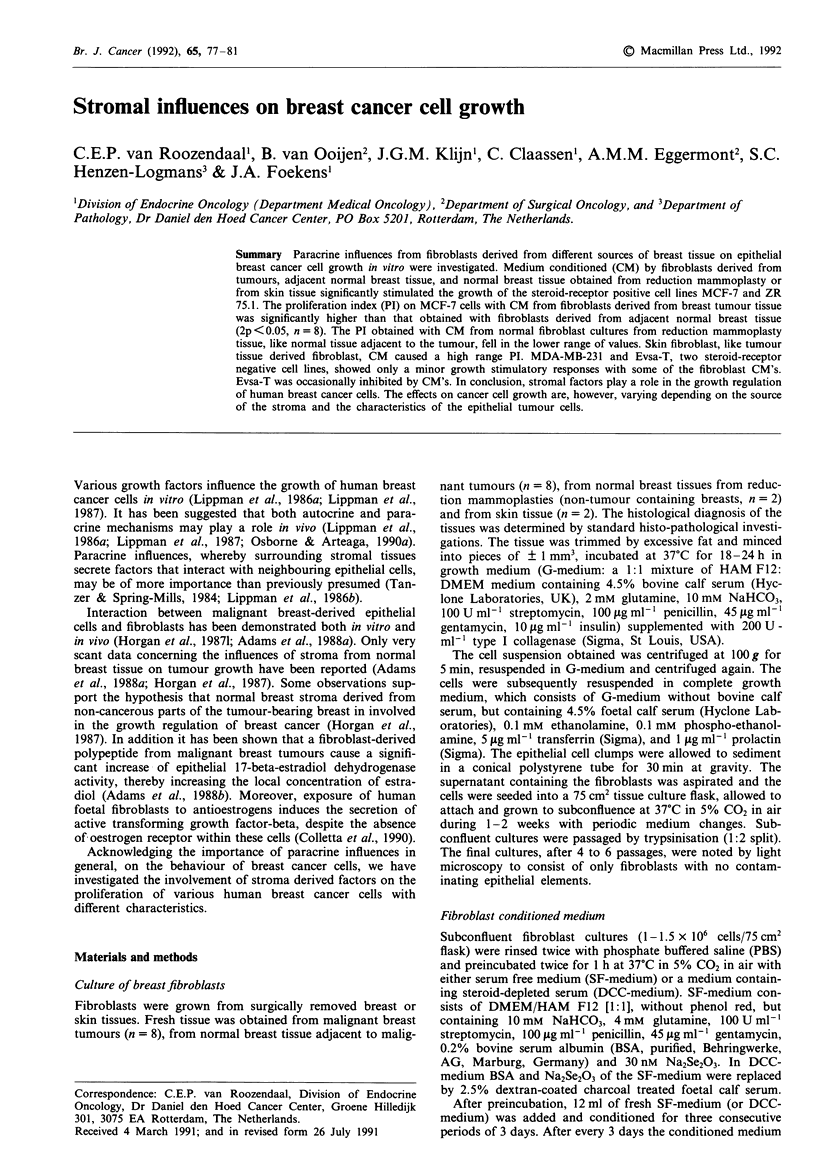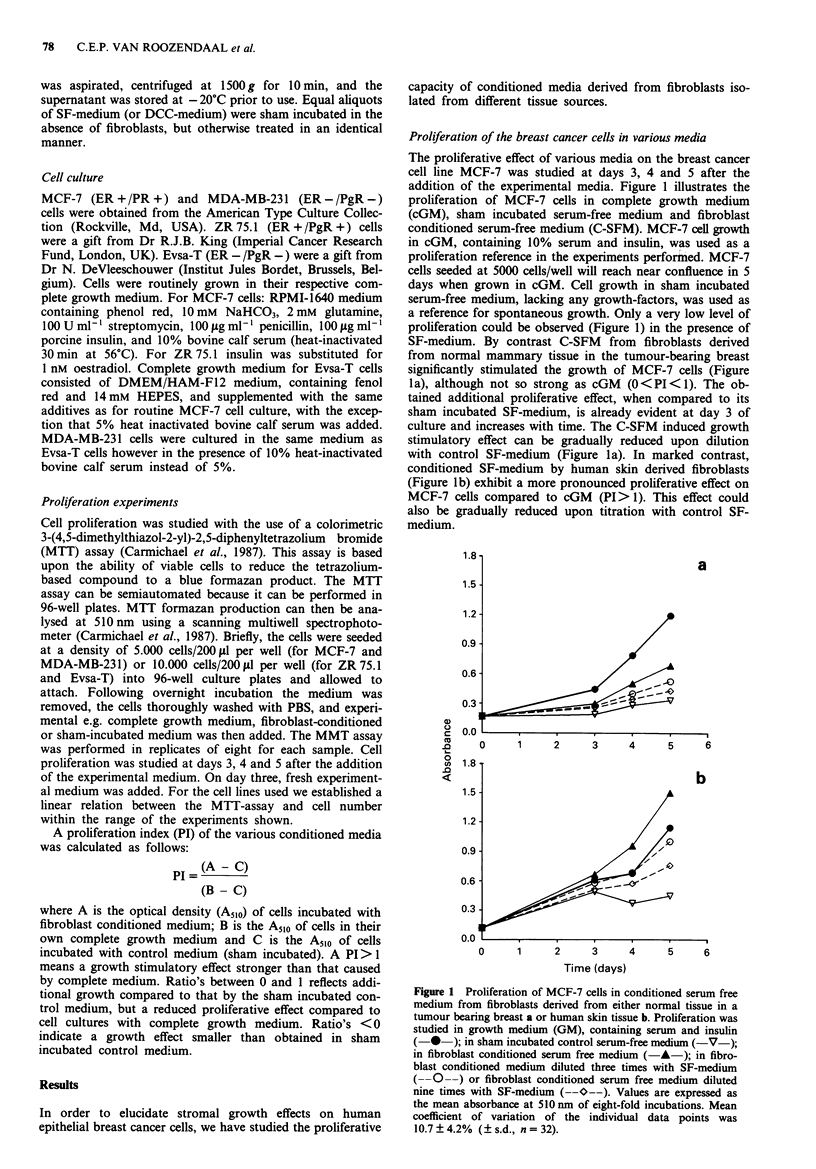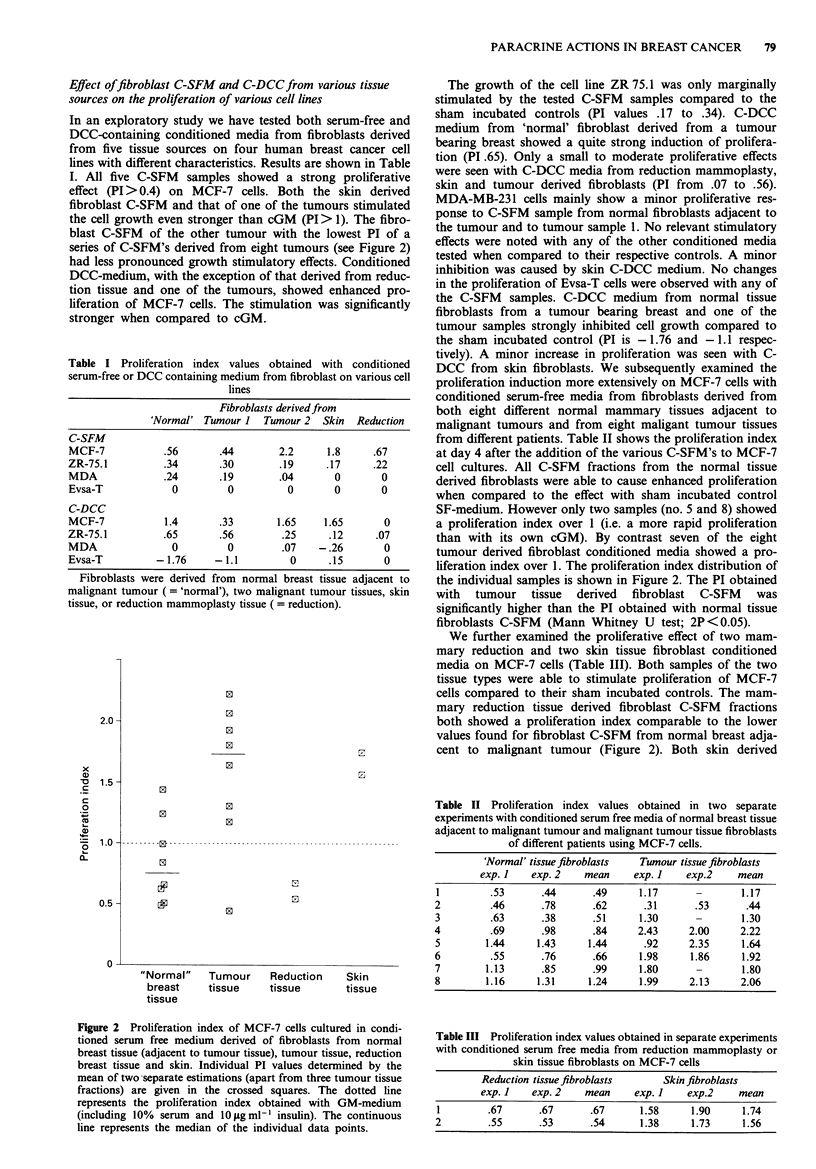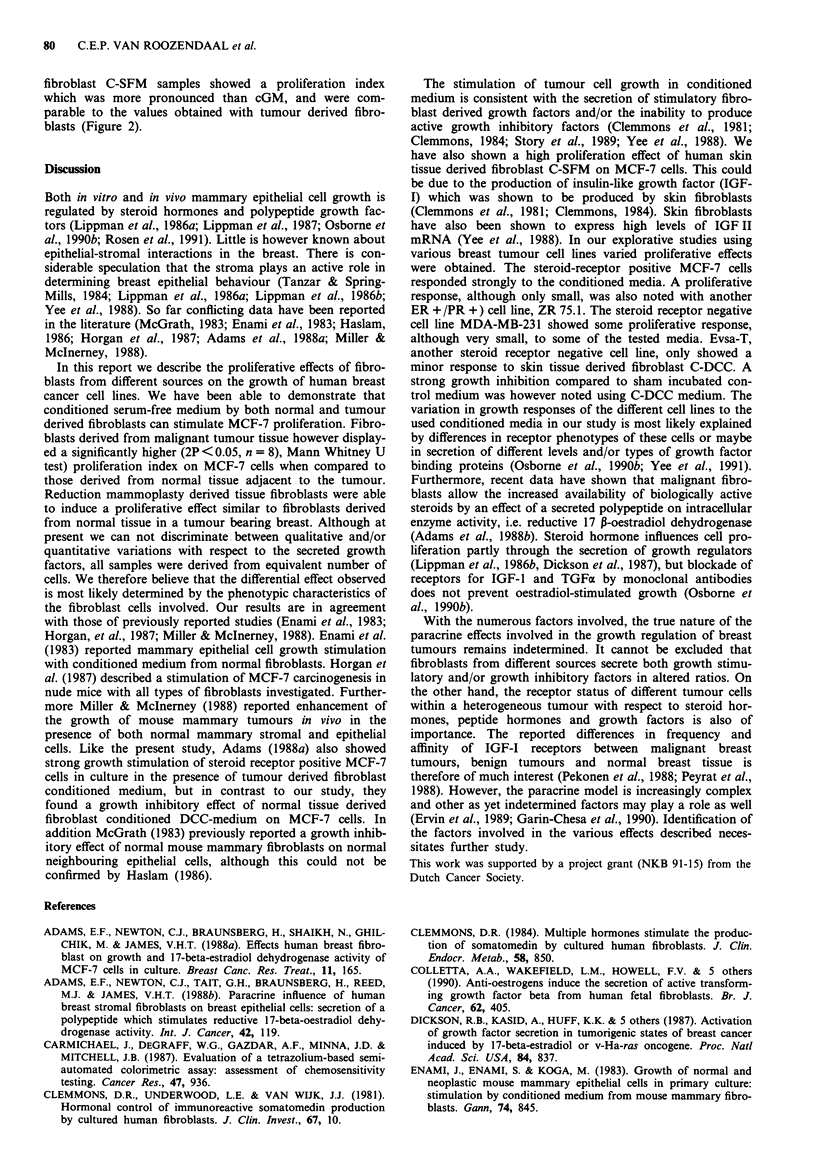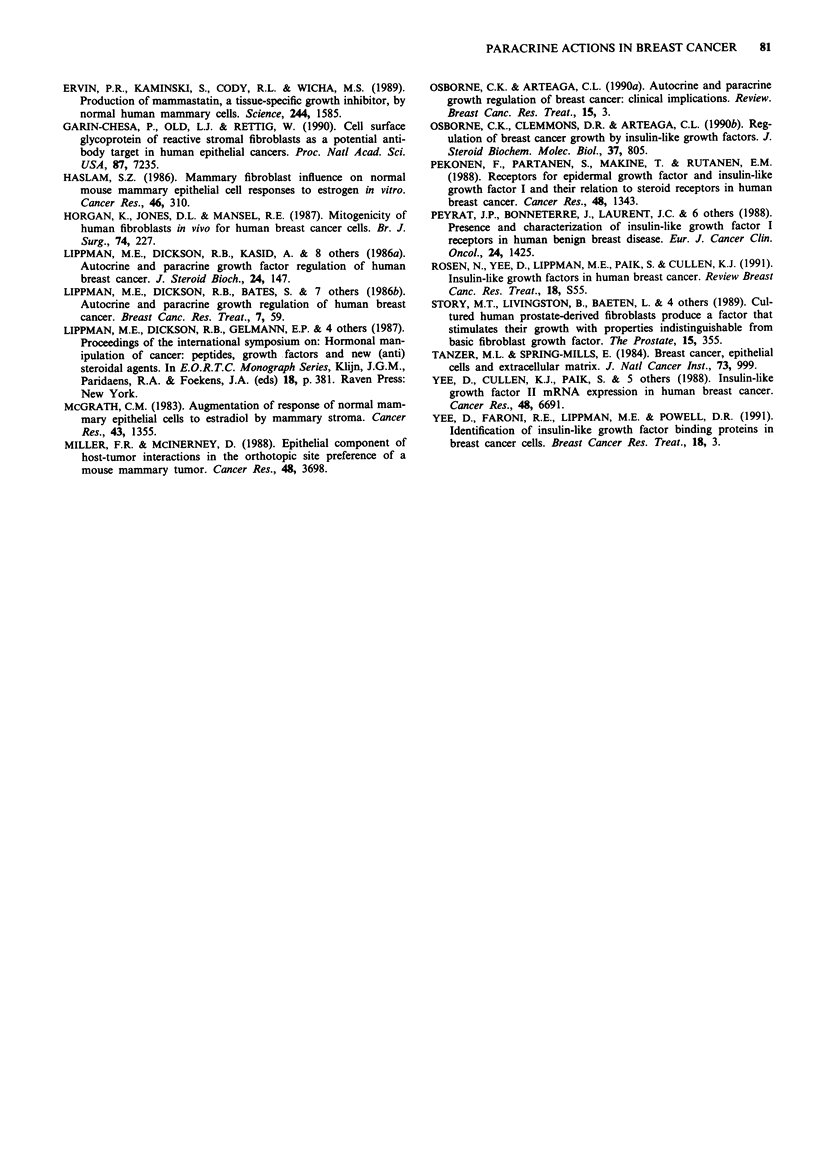# Stromal influences on breast cancer cell growth.

**DOI:** 10.1038/bjc.1992.14

**Published:** 1992-01

**Authors:** C. E. van Roozendaal, B. van Ooijen, J. G. Klijn, C. Claassen, A. M. Eggermont, S. C. Henzen-Logmans, J. A. Foekens

**Affiliations:** Division of Endocrine Oncology (Department Medical Oncology), Dr Daniel den Hoed Cancer Center, Rotterdam, The Netherlands.

## Abstract

Paracrine influences from fibroblasts derived from different sources of breast tissue on epithelial breast cancer cell growth in vitro were investigated. Medium conditioned (CM) by fibroblasts derived from tumours, adjacent normal breast tissue, and normal breast tissue obtained from reduction mammoplasty or from skin tissue significantly stimulated the growth of the steroid-receptor positive cell lines MCF-7 and ZR 75.1. The proliferation index (PI) on MCF-7 cells with CM from fibroblasts derived from breast tumour tissue was significantly higher than that obtained with fibroblasts derived from adjacent normal breast tissue (2p less than 0.05, n = 8). The PI obtained with CM from normal fibroblast cultures from reduction mammoplasty tissue, like normal tissue adjacent to the tumour, fell in the lower range of values. Skin fibroblast, like tumour tissue derived fibroblast, CM caused a high range PI. MDA-MB-231 and Evsa-T, two steroid-receptor negative cell lines, showed only a minor growth stimulatory responses with some of the fibroblast CM's. Evsa-T was occasionally inhibited by CM's. In conclusion, stromal factors play a role in the growth regulation of human breast cancer cells. The effects on cancer cell growth are, however, varying depending on the source of the stroma and the characteristics of the epithelial tumour cells.


Br. J. Cancer (1992), 65, 77-81 © Macmillan Press Ltd., 1992

### Stromal influences on breast cancer cell growth

C.E.P. van Roozendaal[1], B. van Ooijen[2], J.G.M. Klijn[1], C. Claassen[1], A.M.M. Eggermont[2], S.C. Henzen-Logmans[3] & J.A. Foekens[1]

[1]*Division of Endocrine Oncology (Department Medical Oncology)*, [2]*Department of Surgical Oncology, and* [3]*Department of Pathology, Dr Daniel den Hoed Cancer Center, PO Box 5201, Rotterdam, The Netherlands.*

**Summary** Paracrine influences from fibroblasts derived from different sources of breast tissue on epithelial breast cancer cell growth *in vitro* were investigated. Medium conditioned (CM) by fibroblasts derived from tumours, adjacent normal breast tissue, and normal breast tissue obtained from reduction mammoplasty or from skin tissue significantly stimulated the growth of the steroid-receptor positive cell lines MCF-7 and ZR 75.1. The proliferation index (PI) on MCF-7 cells with CM from fibroblasts derived from breast tumour tissue was significantly higher than that obtained with fibroblasts derived from adjacent normal breast tissue ($2p < 0.05$, $n = 8$). The PI obtained with CM from normal fibroblast cultures from reduction mammoplasty tissue, like normal tissue adjacent to the tumour, fell in the lower range of values. Skin fibroblast, like tumour tissue derived fibroblast, CM caused a high range PI. MDA-MB-231 and Evsa-T, two steroid-receptor negative cell lines, showed only a minor growth stimulatory responses with some of the fibroblast CM's. Evsa-T was occasionally inhibited by CM's. In conclusion, stromal factors play a role in the growth regulation of human breast cancer cells. The effects on cancer cell growth are, however, varying depending on the source of the stroma and the characteristics of the epithelial tumour cells.

Various growth factors influence the growth of human breast cancer cells *in vitro* (Lippman *et al.*, 1986*a*; Lippman *et al.*, 1987). It has been suggested that both autocrine and paracrine mechanisms may play a role *in vivo* (Lippman *et al.*, 1986*a*; Lippman *et al.*, 1987; Osborne & Arteaga, 1990*a*). Paracrine influences, whereby surrounding stromal tissues secrete factors that interact with neighbouring epithelial cells, may be of more importance than previously presumed (Tanzer & Spring-Mills, 1984; Lippman *et al.*, 1986*b*).

Interaction between malignant breast-derived epithelial cells and fibroblasts has been demonstrated both *in vitro* and *in vivo* (Horgan *et al.*, 1987l; Adams *et al.*, 1988*a*). Only very scant data concerning the influences of stroma from normal breast tissue on tumour growth have been reported (Adams *et al.*, 1988*a*; Horgan *et al.*, 1987). Some observations support the hypothesis that normal breast stroma derived from non-cancerous parts of the tumour-bearing breast in involved in the growth regulation of breast cancer (Horgan *et al.*, 1987). In addition it has been shown that a fibroblast-derived polypeptide from malignant breast tumours cause a significant increase of epithelial 17-beta-estradiol dehydrogenase activity, thereby increasing the local concentration of estradiol (Adams *et al.*, 1988*b*). Moreover, exposure of human foetal fibroblasts to antioestrogens induces the secretion of active transforming growth factor-beta, despite the absence of oestrogen receptor within these cells (Colletta *et al.*, 1990).

Acknowledging the importance of paracrine influences in general, on the behaviour of breast cancer cells, we have investigated the involvement of stroma derived factors on the proliferation of various human breast cancer cells with different characteristics.

### Materials and methods

### *Culture of breast fibroblasts*

Fibroblasts were grown from surgically removed breast or skin tissues. Fresh tissue was obtained from malignant breast tumours ($n = 8$), from normal breast tissue adjacent to malignant tumours ($n = 8$), from normal breast tissues from reduction mammoplasties (non-tumour containing breasts, $n = 2$) and from skin tissue ($n = 2$). The histological diagnosis of the tissues was determined by standard histo-pathological investigations. The tissue was trimmed by excessive fat and minced into pieces of $\pm 1\ mm^3$, incubated at 37°C for 18–24 h in growth medium (G-medium: a 1:1 mixture of HAM F12: DMEM medium containing 4.5% bovine calf serum (Hyclone Laboratories, UK), 2 mM glutamine, 10 mM $NaHCO_3$, $100\ U\ ml^{-1}$ streptomycin, $100\ \mu g\ ml^{-1}$ penicillin, $45\ \mu g\ ml^{-1}$ gentamycin, $10\ \mu g\ ml^{-1}$ insulin) supplemented with 200 U $ml^{-1}$ type I collagenase (Sigma, St Louis, USA).

The cell suspension obtained was centrifuged at 100 *g* for 5 min, resuspended in G-medium and centrifuged again. The cells were subsequently resuspended in complete growth medium, which consists of G-medium without bovine calf serum, but containing 4.5% foetal calf serum (Hyclone Laboratories), 0.1 mM ethanolamine, 0.1 mM phospho-ethanolamine, $5\ \mu g\ ml^{-1}$ transferrin (Sigma), and $1\ \mu g\ ml^{-1}$ prolactin (Sigma). The epithelial cell clumps were allowed to sediment in a conical polystyrene tube for 30 min at gravity. The supernatant containing the fibroblasts was aspirated and the cells were seeded into a $75\ cm^2$ tissue culture flask, allowed to attach and grown to subconfluence at 37°C in 5% $CO_2$ in air during 1–2 weeks with periodic medium changes. Subconfluent cultures were passaged by trypsinisation (1:2 split). The final cultures, after 4 to 6 passages, were noted by light microscopy to consist of only fibroblasts with no contaminating epithelial elements.

### *Fibroblast conditioned medium*

Subconfluent fibroblast cultures ($1-1.5 \times 10^6$ cells/75 $cm^2$ flask) were rinsed twice with phosphate buffered saline (PBS) and preincubated twice for 1 h at 37°C in 5% $CO_2$ in air with either serum free medium (SF-medium) or a medium containing steroid-depleted serum (DCC-medium). SF-medium consists of DMEM/HAM F12 [1:1], without phenol red, but containing 10 mM $NaHCO_3$, 4 mM glutamine, $100\ U\ ml^{-1}$ streptomycin, $100\ \mu g\ ml^{-1}$ penicillin, $45\ \mu g\ ml^{-1}$ gentamycin, 0.2% bovine serum albumin (BSA, purified, Behringwerke, AG, Marburg, Germany) and 30 nM $Na_2Se_2O_3$. In DCC-medium BSA and $Na_2Se_2O_3$ of the SF-medium were replaced by 2.5% dextran-coated charcoal treated foetal calf serum.

After preincubation, 12 ml of fresh SF-medium (or DCC-medium) was added and conditioned for three consecutive periods of 3 days. After every 3 days the conditioned medium

Correspondence: C.E.P. van Roozendaal, Division of Endocrine Oncology, Dr Daniel den Hoed Cancer Center, Groene Hilledijk 301, 3075 EA Rotterdam, The Netherlands.
Received 4 March 1991; and in revised form 26 July 1991

was aspirated, centrifuged at 1500 g for 10 min, and the supernatant was stored at −20°C prior to use. Equal aliquots of SF-medium (or DCC-medium) were sham incubated in the absence of fibroblasts, but otherwise treated in an identical manner.

*Cell culture*

MCF-7 (ER+/PR+) and MDA-MB-231 (ER−/PgR−) cells were obtained from the American Type Culture Collection (Rockville, Md, USA). ZR 75.1 (ER+/PgR+) cells were a gift from Dr R.J.B. King (Imperial Cancer Research Fund, London, UK). Evsa-T (ER−/PgR−) were a gift from Dr N. DeVleeschouwer (Institut Jules Bordet, Brussels, Belgium). Cells were routinely grown in their respective complete growth medium. For MCF-7 cells: RPMI-1640 medium containing phenol red, 10 mM $NaHCO_3$, 2 mM glutamine, 100 U $ml^{-1}$ streptomycin, 100 μg $ml^{-1}$ penicillin, 100 μg $ml^{-1}$ porcine insulin, and 10% bovine calf serum (heat-inactivated 30 min at 56°C). For ZR 75.1 insulin was substituted for 1 nM oestradiol. Complete growth medium for Evsa-T cells consisted of DMEM/HAM-F12 medium, containing fenol red and 14 mM HEPES, and supplemented with the same additives as for routine MCF-7 cell culture, with the exception that 5% heat inactivated bovine calf serum was added. MDA-MB-231 cells were cultured in the same medium as Evsa-T cells however in the presence of 10% heat-inactivated bovine calf serum instead of 5%.

*Proliferation experiments*

Cell proliferation was studied with the use of a colorimetric 3-(4,5-dimethylthiazol-2-yl)-2,5-diphenyltetrazolium bromide (MTT) assay (Carmichael *et al.*, 1987). This assay is based upon the ability of viable cells to reduce the tetrazolium-based compound to a blue formazan product. The MTT assay can be semiautomated because it can be performed in 96-well plates. MTT formazan production can then be analysed at 510 nm using a scanning multiwell spectrophotometer (Carmichael *et al.*, 1987). Briefly, the cells were seeded at a density of 5.000 cells/200 μl per well (for MCF-7 and MDA-MB-231) or 10.000 cells/200 μl per well (for ZR 75.1 and Evsa-T) into 96-well culture plates and allowed to attach. Following overnight incubation the medium was removed, the cells thoroughly washed with PBS, and experimental e.g. complete growth medium, fibroblast-conditioned or sham-incubated medium was then added. The MMT assay was performed in replicates of eight for each sample. Cell proliferation was studied at days 3, 4 and 5 after the addition of the experimental medium. On day three, fresh experimental medium was added. For the cell lines used we established a linear relation between the MTT-assay and cell number within the range of the experiments shown.

A proliferation index (PI) of the various conditioned media was calculated as follows:

$$PI = \frac{(A - C)}{(B - C)}$$

where A is the optical density ($A_{510}$) of cells incubated with fibroblast conditioned medium; B is the $A_{510}$ of cells in their own complete growth medium and C is the $A_{510}$ of cells incubated with control medium (sham incubated). A $PI > 1$ means a growth stimulatory effect stronger than that caused by complete medium. Ratio's between 0 and 1 reflects additional growth compared to that by the sham incubated control medium, but a reduced proliferative effect compared to cell cultures with complete growth medium. Ratio's $< 0$ indicate a growth effect smaller than obtained in sham incubated control medium.

### Results

In order to elucidate stromal growth effects on human epithelial breast cancer cells, we have studied the proliferative capacity of conditioned media derived from fibroblasts isolated from different tissue sources.

*Proliferation of the breast cancer cells in various media*

The proliferative effect of various media on the breast cancer cell line MCF-7 was studied at days 3, 4 and 5 after the addition of the experimental media. Figure 1 illustrates the proliferation of MCF-7 cells in complete growth medium (cGM), sham incubated serum-free medium and fibroblast conditioned serum-free medium (C-SFM). MCF-7 cell growth in cGM, containing 10% serum and insulin, was used as a proliferation reference in the experiments performed. MCF-7 cells seeded at 5000 cells/well will reach near confluence in 5 days when grown in cGM. Cell growth in sham incubated serum-free medium, lacking any growth-factors, was used as a reference for spontaneous growth. Only a very low level of proliferation could be observed (Figure 1) in the presence of SF-medium. By contrast C-SFM from fibroblasts derived from normal mammary tissue in the tumour-bearing breast significantly stimulated the growth of MCF-7 cells (Figure 1a), although not so strong as cGM ($0 < PI < 1$). The obtained additional proliferative effect, when compared to its sham incubated SF-medium, is already evident at day 3 of culture and increases with time. The C-SFM induced growth stimulatory effect can be gradually reduced upon dilution with control SF-medium (Figure 1a). In marked contrast, conditioned SF-medium by human skin derived fibroblasts (Figure 1b) exhibit a more pronounced proliferative effect on MCF-7 cells compared to cGM ($PI > 1$). This effect could also be gradually reduced upon titration with control SF-medium.

**Figure 1** Proliferation of MCF-7 cells in conditioned serum free medium from fibroblasts derived from either normal tissue in a tumour bearing breast **a** or human skin tissue **b**. Proliferation was studied in growth medium (GM), containing serum and insulin (—●—); in sham incubated control serum-free medium (—▽—); in fibroblast conditioned serum free medium (—▲—); in fibroblast conditioned medium diluted three times with SF-medium (--○--) or fibroblast conditioned serum free medium diluted nine times with SF-medium (--◇--). Values are expressed as the mean absorbance at 510 nm of eight-fold incubations. Mean coefficient of variation of the individual data points was 10.7 ± 4.2% (± s.d., *n* = 32).

*Effect of fibroblast C-SFM and C-DCC from various tissue sources on the proliferation of various cell lines*

In an exploratory study we have tested both serum-free and DCC-containing conditioned media from fibroblasts derived from five tissue sources on four human breast cancer cell lines with different characteristics. Results are shown in Table I. All five C-SFM samples showed a strong proliferative effect (PI > 0.4) on MCF-7 cells. Both the skin derived fibroblast C-SFM and that of one of the tumours stimulated the cell growth even stronger than cGM (PI > 1). The fibroblast C-SFM of the other tumour with the lowest PI of a series of C-SFM's derived from eight tumours (see Figure 2) had less pronounced growth stimulatory effects. Conditioned DCC-medium, with the exception of that derived from reduction tissue and one of the tumours, showed enhanced proliferation of MCF-7 cells. The stimulation was significantly stronger when compared to cGM.

**Table I** Proliferation index values obtained with conditioned serum-free or DCC containing medium from fibroblast on various cell lines

| | *Fibroblasts derived from* | | | | |
|---|---|---|---|---|---|
| | *'Normal'* | *Tumour 1* | *Tumour 2* | *Skin* | *Reduction* |
| *C-SFM* | | | | | |
| MCF-7 | .56 | .44 | 2.2 | 1.8 | .67 |
| ZR-75.1 | .34 | .30 | .19 | .17 | .22 |
| MDA | .24 | .19 | .04 | 0 | 0 |
| Evsa-T | 0 | 0 | 0 | 0 | 0 |
| *C-DCC* | | | | | |
| MCF-7 | 1.4 | .33 | 1.65 | 1.65 | 0 |
| ZR-75.1 | .65 | .56 | .25 | .12 | .07 |
| MDA | 0 | 0 | .07 | −.26 | 0 |
| Evsa-T | −1.76 | −1.1 | 0 | .15 | 0 |

Fibroblasts were derived from normal breast tissue adjacent to malignant tumour ( = 'normal'), two malignant tumour tissues, skin tissue, or reduction mammoplasty tissue ( = reduction).

**Figure 2** Proliferation index of MCF-7 cells cultured in conditioned serum free medium derived of fibroblasts from normal breast tissue (adjacent to tumour tissue), tumour tissue, reduction breast tissue and skin. Individual PI values determined by the mean of two separate estimations (apart from three tumour tissue fractions) are given in the crossed squares. The dotted line represents the proliferation index obtained with GM-medium (including 10% serum and 10 $\mu g\,ml^{-1}$ insulin). The continuous line represents the median of the individual data points.

The growth of the cell line ZR 75.1 was only marginally stimulated by the tested C-SFM samples compared to the sham incubated controls (PI values .17 to .34). C-DCC medium from 'normal' fibroblast derived from a tumour bearing breast showed a quite strong induction of proliferation (PI .65). Only a small to moderate proliferative effects were seen with C-DCC media from reduction mammoplasty, skin and tumour derived fibroblasts (PI from .07 to .56). MDA-MB-231 cells mainly show a minor proliferative response to C-SFM sample from normal fibroblasts adjacent to the tumour and to tumour sample 1. No relevant stimulatory effects were noted with any of the other conditioned media tested when compared to their respective controls. A minor inhibition was caused by skin C-DCC medium. No changes in the proliferation of Evsa-T cells were observed with any of the C-SFM samples. C-DCC medium from normal tissue fibroblasts from a tumour bearing breast and one of the tumour samples strongly inhibited cell growth compared to the sham incubated control (PI is −1.76 and −1.1 respectively). A minor increase in proliferation was seen with C-DCC from skin fibroblasts. We subsequently examined the proliferation induction more extensively on MCF-7 cells with conditioned serum-free media from fibroblasts derived from both eight different normal mammary tissues adjacent to malignant tumours and from eight maligant tumour tissues from different patients. Table II shows the proliferation index at day 4 after the addition of the various C-SFM's to MCF-7 cell cultures. All C-SFM fractions from the normal tissue derived fibroblasts were able to cause enhanced proliferation when compared to the effect with sham incubated control SF-medium. However only two samples (no. 5 and 8) showed a proliferation index over 1 (i.e. a more rapid proliferation than with its own cGM). By contrast seven of the eight tumour derived fibroblast conditioned media showed a proliferation index over 1. The proliferation index distribution of the individual samples is shown in Figure 2. The PI obtained with tumour tissue derived fibroblast C-SFM was significantly higher than the PI obtained with normal tissue fibroblasts C-SFM (Mann Whitney U test; $2P < 0.05$).

We further examined the proliferative effect of two mammary reduction and two skin tissue fibroblast conditioned media on MCF-7 cells (Table III). Both samples of the two tissue types were able to stimulate proliferation of MCF-7 cells compared to their sham incubated controls. The mammary reduction tissue derived fibroblast C-SFM fractions both showed a proliferation index comparable to the lower values found for fibroblast C-SFM from normal breast adjacent to malignant tumour (Figure 2). Both skin derived

**Table II** Proliferation index values obtained in two separate experiments with conditioned serum free media of normal breast tissue adjacent to malignant tumour and malignant tumour tissue fibroblasts of different patients using MCF-7 cells.

| | *'Normal' tissue fibroblasts* | | | *Tumour tissue fibroblasts* | | |
|---|---|---|---|---|---|---|
| | *exp. 1* | *exp. 2* | *mean* | *exp. 1* | *exp.2* | *mean* |
| 1 | .53 | .44 | .49 | 1.17 | – | 1.17 |
| 2 | .46 | .78 | .62 | .31 | .53 | .44 |
| 3 | .63 | .38 | .51 | 1.30 | – | 1.30 |
| 4 | .69 | .98 | .84 | 2.43 | 2.00 | 2.22 |
| 5 | 1.44 | 1.43 | 1.44 | .92 | 2.35 | 1.64 |
| 6 | .55 | .76 | .66 | 1.98 | 1.86 | 1.92 |
| 7 | 1.13 | .85 | .99 | 1.80 | – | 1.80 |
| 8 | 1.16 | 1.31 | 1.24 | 1.99 | 2.13 | 2.06 |

**Table III** Proliferation index values obtained in separate experiments with conditioned serum free media from reduction mammoplasty or skin tissue fibroblasts on MCF-7 cells

| | *Reduction tissue fibroblasts* | | | *Skin fibroblasts* | | |
|---|---|---|---|---|---|---|
| | *exp. 1* | *exp. 2* | *mean* | *exp. 1* | *exp.2* | *mean* |
| 1 | .67 | .67 | .67 | 1.58 | 1.90 | 1.74 |
| 2 | .55 | .53 | .54 | 1.38 | 1.73 | 1.56 |

fibroblast C-SFM samples showed a proliferation index which was more pronounced than cGM, and were comparable to the values obtained with tumour derived fibroblasts (Figure 2).

### Discussion

Both *in vitro* and *in vivo* mammary epithelial cell growth is regulated by steroid hormones and polypeptide growth factors (Lippman *et al.*, 1986*a*; Lippman *et al.*, 1987; Osborne *et al.*, 1990*b*; Rosen *et al.*, 1991). Little is however known about epithelial-stromal interactions in the breast. There is considerable speculation that the stroma plays an active role in determining breast epithelial behaviour (Tanzar & Spring-Mills, 1984; Lippman *et al.*, 1986*a*; Lippman *et al.*, 1986*b*; Yee *et al.*, 1988). So far conflicting data have been reported in the literature (McGrath, 1983; Enami *et al.*, 1983; Haslam, 1986; Horgan *et al.*, 1987; Adams *et al.*, 1988*a*; Miller & McInerney, 1988).

In this report we describe the proliferative effects of fibroblasts from different sources on the growth of human breast cancer cell lines. We have been able to demonstrate that conditioned serum-free medium by both normal and tumour derived fibroblasts can stimulate MCF-7 proliferation. Fibroblasts derived from malignant tumour tissue however displayed a significantly higher ($2P < 0.05$, $n = 8$), Mann Whitney U test) proliferation index on MCF-7 cells when compared to those derived from normal tissue adjacent to the tumour. Reduction mammoplasty derived tissue fibroblasts were able to induce a proliferative effect similar to fibroblasts derived from normal tissue in a tumour bearing breast. Although at present we can not discriminate between qualitative and/or quantitative variations with respect to the secreted growth factors, all samples were derived from equivalent number of cells. We therefore believe that the differential effect observed is most likely determined by the phenotypic characteristics of the fibroblast cells involved. Our results are in agreement with those of previously reported studies (Enami *et al.*, 1983; Horgan, *et al.*, 1987; Miller & McInerney, 1988). Enami *et al.* (1983) reported mammary epithelial cell growth stimulation with conditioned medium from normal fibroblasts. Horgan *et al.* (1987) described a stimulation of MCF-7 carcinogenesis in nude mice with all types of fibroblasts investigated. Furthermore Miller & McInerney (1988) reported enhancement of the growth of mouse mammary tumours *in vivo* in the presence of both normal mammary stromal and epithelial cells. Like the present study, Adams (1988*a*) also showed strong growth stimulation of steroid receptor positive MCF-7 cells in culture in the presence of tumour derived fibroblast conditioned medium, but in contrast to our study, they found a growth inhibitory effect of normal tissue derived fibroblast conditioned DCC-medium on MCF-7 cells. In addition McGrath (1983) previously reported a growth inhibitory effect of normal mouse mammary fibroblasts on normal neighbouring epithelial cells, although this could not be confirmed by Haslam (1986).

The stimulation of tumour cell growth in conditioned medium is consistent with the secretion of stimulatory fibroblast derived growth factors and/or the inability to produce active growth inhibitory factors (Clemmons *et al.*, 1981; Clemmons, 1984; Story *et al.*, 1989; Yee *et al.*, 1988). We have also shown a high proliferation effect of human skin tissue derived fibroblast C-SFM on MCF-7 cells. This could be due to the production of insulin-like growth factor (IGF-I) which was shown to be produced by skin fibroblasts (Clemmons *et al.*, 1981; Clemmons, 1984). Skin fibroblasts have also been shown to express high levels of IGF II mRNA (Yee *et al.*, 1988). In our explorative studies using various breast tumour cell lines varied proliferative effects were obtained. The steroid-receptor positive MCF-7 cells responded strongly to the conditioned media. A proliferative response, although only small, was also noted with another ER +/PR +) cell line, ZR 75.1. The steroid receptor negative cell line MDA-MB-231 showed some proliferative response, although very small, to some of the tested media. Evsa-T, another steroid receptor negative cell line, only showed a minor response to skin tissue derived fibroblast C-DCC. A strong growth inhibition compared to sham incubated control medium was however noted using C-DCC medium. The variation in growth responses of the different cell lines to the used conditioned media in our study is most likely explained by differences in receptor phenotypes of these cells or maybe in secretion of different levels and/or types of growth factor binding proteins (Osborne *et al.*, 1990*b*; Yee *et al.*, 1991). Furthermore, recent data have shown that malignant fibroblasts allow the increased availability of biologically active steroids by an effect of a secreted polypeptide on intracellular enzyme activity, i.e. reductive 17 $\beta$-oestradiol dehydrogenase (Adams *et al.*, 1988*b*). Steroid hormone influences cell proliferation partly through the secretion of growth regulators (Lippman *et al.*, 1986*b*, Dickson *et al.*, 1987), but blockade of receptors for IGF-1 and TGF$\alpha$ by monoclonal antibodies does not prevent oestradiol-stimulated growth (Osborne *et al.*, 1990*b*).

With the numerous factors involved, the true nature of the paracrine effects involved in the growth regulation of breast tumours remains indetermined. It cannot be excluded that fibroblasts from different sources secrete both growth stimulatory and/or growth inhibitory factors in altered ratios. On the other hand, the receptor status of different tumour cells within a heterogeneous tumour with respect to steroid hormones, peptide hormones and growth factors is also of importance. The reported differences in frequency and affinity of IGF-I receptors between malignant breast tumours, benign tumours and normal breast tissue is therefore of much interest (Pekonen *et al.*, 1988; Peyrat *et al.*, 1988). However, the paracrine model is increasingly complex and other as yet indetermined factors may play a role as well (Ervin *et al.*, 1989; Garin-Chesa *et al.*, 1990). Identification of the factors involved in the various effects described necessitates further study.

This work was supported by a project grant (NKB 91-15) from the Dutch Cancer Society.

### References

ADAMS, E.F., NEWTON, C.J., BRAUNSBERG, H., SHAIKH, N., GHILCHIK, M. & JAMES, V.H.T. (1988*a*). Effects human breast fibroblast on growth and 17-beta-estradiol dehydrogenase activity of MCF-7 cells in culture. *Breast Canc. Res. Treat.*, **11**, 165.

ADAMS, E.F., NEWTON, C.J., TAIT, G.H., BRAUNSBERG, H., REED, M.J. & JAMES, V.H.T. (1988*b*). Paracrine influence of human breast stromal fibroblasts on breast epithelial cells: secretion of a polypeptide which stimulates reductive 17-beta-oestradiol dehydrogenase activity. *Int. J. Cancer*, **42**, 119.

CARMICHAEL, J., DEGRAFF, W.G., GAZDAR, A.F., MINNA, J.D. & MITCHELL, J.B. (1987). Evaluation of a tetrazolium-based semiautomated colorimetric assay: assessment of chemosensitivity testing. *Cancer Res.*, **47**, 936.

CLEMMONS, D.R., UNDERWOOD, L.E. & VAN WIJK, J.J. (1981). Hormonal control of immunoreactive somatomedin production by cultured human fibroblasts. *J. Clin. Invest.*, **67**, 10.

CLEMMONS, D.R. (1984). Multiple hormones stimulate the production of somatomedin by cultured human fibroblasts. *J. Clin. Endocr. Metab.*, **58**, 850.

COLLETTA, A.A., WAKEFIELD, L.M., HOWELL, F.V. & 5 others (1990). Anti-oestrogens induce the secretion of active transforming growth factor beta from human fetal fibroblasts. *Br. J. Cancer*, **62**, 405.

DICKSON, R.B., KASID, A., HUFF, K.K. & 5 others (1987). Activation of growth factor secretion in tumorigenic states of breast cancer induced by 17-beta-estradiol or v-Ha-*ras* oncogene. *Proc. Natl Acad. Sci. USA*, **84**, 837.

ENAMI, J., ENAMI, S. & KOGA, M. (1983). Growth of normal and neoplastic mouse mammary epithelial cells in primary culture: stimulation by conditioned medium from mouse mammary fibroblasts. *Gann*, **74**, 845.

ERVIN, P.R., KAMINSKI, S., CODY, R.L. & WICHA, M.S. (1989). Production of mammastatin, a tissue-specific growth inhibitor, by normal human mammary cells. *Science*, **244**, 1585.

GARIN-CHESA, P., OLD, L.J. & RETTIG, W. (1990). Cell surface glycoprotein of reactive stromal fibroblasts as a potential antibody target in human epithelial cancers. *Proc. Natl Acad. Sci. USA*, **87**, 7235.

HASLAM, S.Z. (1986). Mammary fibroblast influence on normal mouse mammary epithelial cell responses to estrogen *in vitro*. *Cancer Res.*, **46**, 310.

HORGAN, K., JONES, D.L. & MANSEL, R.E. (1987). Mitogenicity of human fibroblasts *in vivo* for human breast cancer cells. *Br. J. Surg.*, **74**, 227.

LIPPMAN, M.E., DICKSON, R.B., KASID, A. & 8 others (1986*a*). Autocrine and paracrine growth factor regulation of human breast cancer. *J. Steroid Bioch.*, **24**, 147.

LIPPMAN, M.E., DICKSON, R.B., BATES, S. & 7 others (1986*b*). Autocrine and paracrine growth regulation of human breast cancer. *Breast Canc. Res. Treat.*, **7**, 59.

LIPPMAN, M.E., DICKSON, R.B., GELMANN, E.P. & 4 others (1987). Proceedings of the international symposium on: Hormonal manipulation of cancer: peptides, growth factors and new (anti) steroidal agents. In *E.O.R.T.C. Monograph Series*, Klijn, J.G.M., Paridaens, R.A. & Foekens, J.A. (eds) **18**, p. 381. Raven Press: New York.

MCGRATH, C.M. (1983). Augmentation of response of normal mammary epithelial cells to estradiol by mammary stroma. *Cancer Res.*, **43**, 1355.

MILLER, F.R. & MCINERNEY, D. (1988). Epithelial component of host-tumor interactions in the orthotopic site preference of a mouse mammary tumor. *Cancer Res.*, **48**, 3698.

OSBORNE, C.K. & ARTEAGA, C.L. (1990*a*). Autocrine and paracrine growth regulation of breast cancer: clinical implications. *Review. Breast Canc. Res. Treat.*, **15**, 3.

OSBORNE, C.K., CLEMMONS, D.R. & ARTEAGA, C.L. (1990*b*). Regulation of breast cancer growth by insulin-like growth factors. *J. Steroid Biochem. Molec. Biol.*, **37**, 805.

PEKONEN, F., PARTANEN, S., MAKINE, T. & RUTANEN, E.M. (1988). Receptors for epidermal growth factor and insulin-like growth factor I and their relation to steroid receptors in human breast cancer. *Cancer Res.*, **48**, 1343.

PEYRAT, J.P., BONNETERRE, J., LAURENT, J.C. & 6 others (1988). Presence and characterization of insulin-like growth factor I receptors in human benign breast disease. *Eur. J. Cancer Clin. Oncol.*, **24**, 1425.

ROSEN, N., YEE, D., LIPPMAN, M.E., PAIK, S. & CULLEN, K.J. (1991). Insulin-like growth factors in human breast cancer. *Review Breast Canc. Res. Treat.*, **18**, S55.

STORY, M.T., LIVINGSTON, B., BAETEN, L. & 4 others (1989). Cultured human prostate-derived fibroblasts produce a factor that stimulates their growth with properties indistinguishable from basic fibroblast growth factor. *The Prostate*, **15**, 355.

TANZER, M.L. & SPRING-MILLS, E. (1984). Breast cancer, epithelial cells and extracellular matrix. *J. Natl Cancer Inst.*, **73**, 999.

YEE, D., CULLEN, K.J., PAIK, S. & 5 others (1988). Insulin-like growth factor II mRNA expression in human breast cancer. *Cancer Res.*, **48**, 6691.

YEE, D., FARONI, R.E., LIPPMAN, M.E. & POWELL, D.R. (1991). Identification of insulin-like growth factor binding proteins in breast cancer cells. *Breast Cancer Res. Treat.*, **18**, 3.